# Supplementary material for: Evaluation of Sample Preservation and Storage Methods for Metaproteomics Analysis of Intestinal Microbiomes
Source: Microbiol Spectr. 2021 Dec 15;9(3):e01877-21. doi: 10.1128/Spectrum.01877-21 (PMC8672883; doi:10.1128/Spectrum.01877-21)
Supplement: SUPPLEMENTAL FILE 1 — Supplemental material. Download SPECTRUM01877-21_Supp_1_seq1.pdf, PDF file, 0.3 MB [file spectrum01877-21_supp_1_seq1.pdf]

Supplementary information for "Evaluation of sample preservation and storage methods for metaproteomics analysis of intestinal microbiomes"

Angie Mordant[1], Manuel Kleiner[1]

Affiliations:

1. Department of Plant and Microbial Biology, North Carolina State University, Raleigh, NC, USA

Correspondence:

Angie Mordant - almordan@ncsu.edu

Manuel Kleiner - mkleine@ncsu.edu

Additional file 1: Quantification data for identified proteins.
Data sheets containing quantitative data for all identified proteins (i) expressed as Peptide Spectrum Matches (PSMs), (ii) expressed as percent Normalized Spectral Abundance Factors (%NSAFs), and (iii) expressed as Centered-Log-Ratio (CLR) transformed values. **(XLSX 7 MB)**

Additional file 2: Differentially abundant proteins.
Data sheet containing protein accessions, -log(p-value), and fold difference for proteins that were significantly different between preservation methods (Student's T-test corrected for multiple hypothesis testing with a permutation-based FDR of 5%, S0=0.1). **(XLSX 142 KB)**

Additional file 3: List of proteins detected per treatment and their corresponding molecular weights and isoelectric points. (XLSX 3 MB)

Additional file 4: Number of predicted transmembrane domains for the proteins identified per treatment.
Data sheet displays the output of the TMHMM 2.0 Server [1] from searching sequences of the identified proteins. **(XLSX 1 MB)**

Additional file 5: Measured microbiome composition in each treatment based on metaproteomes.
Taxonomic composition at the (i) phylum and at the (ii) genus level. **(XLSX 15 KB)**

SUPPLEMENTARY RESULTS

For each treatment, we plotted the relative abundance of each protein, expressed as percent Normalized Spectral Abundance Factors (%NSAFs, [2]) between every replicate pair of samples. This showed that within-treatment variability was low**.** Figures S1 - S6 are scatterplot matrices showing the linear correlations of replicates and the corresponding Pearson correlation coefficients. We prepared the plots in R (version 4. 0. 2; psych_2.1.3 package).

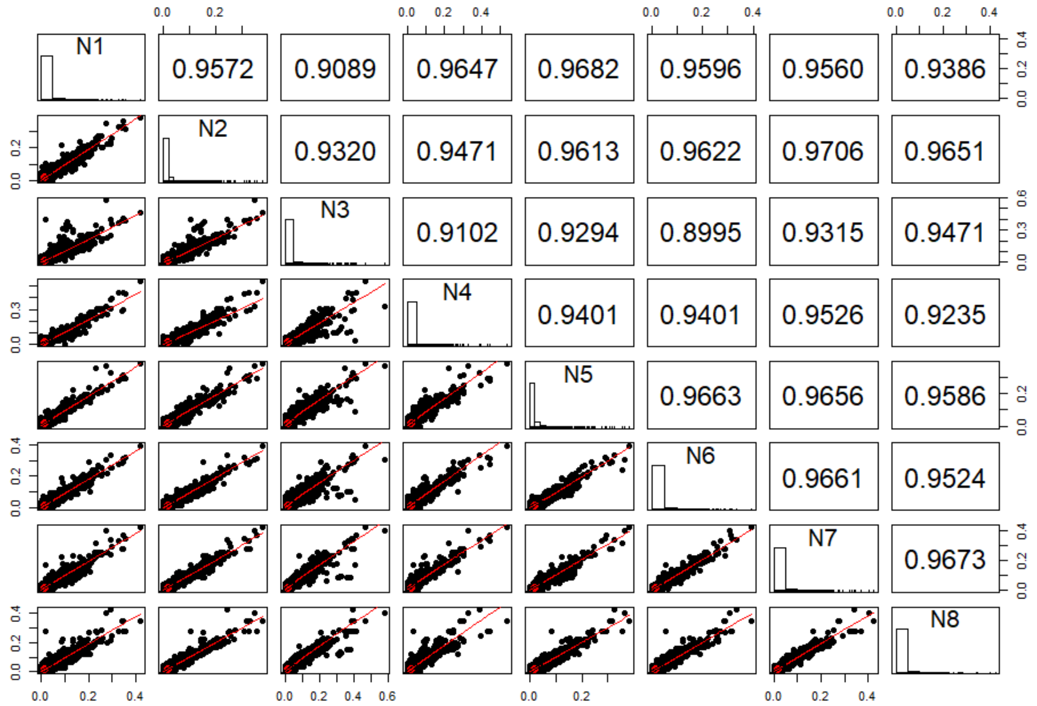

Figure S1. Linear correlation of NAP buffer (N) replicates. Plots are displayed in the lower panel and the corresponding Pearson correlation coefficients are displayed in the upper panel.

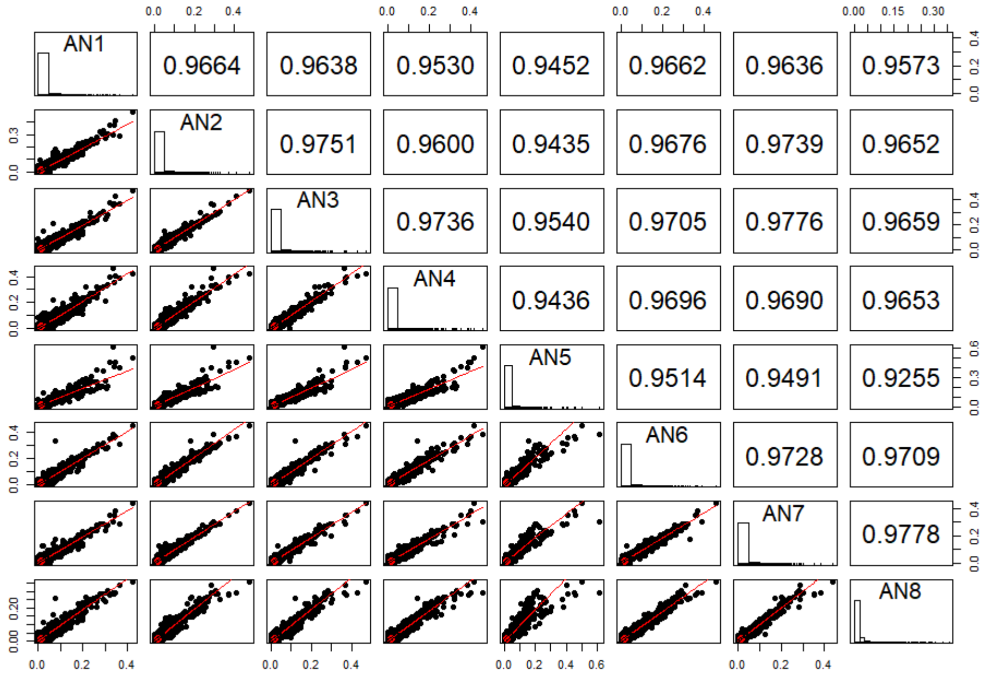

Figure S2. Linear correlation of Autoclaved NAP buffer (AN) replicates. Plots are displayed in the lower panel and the corresponding Pearson correlation coefficients are displayed in the upper panel.

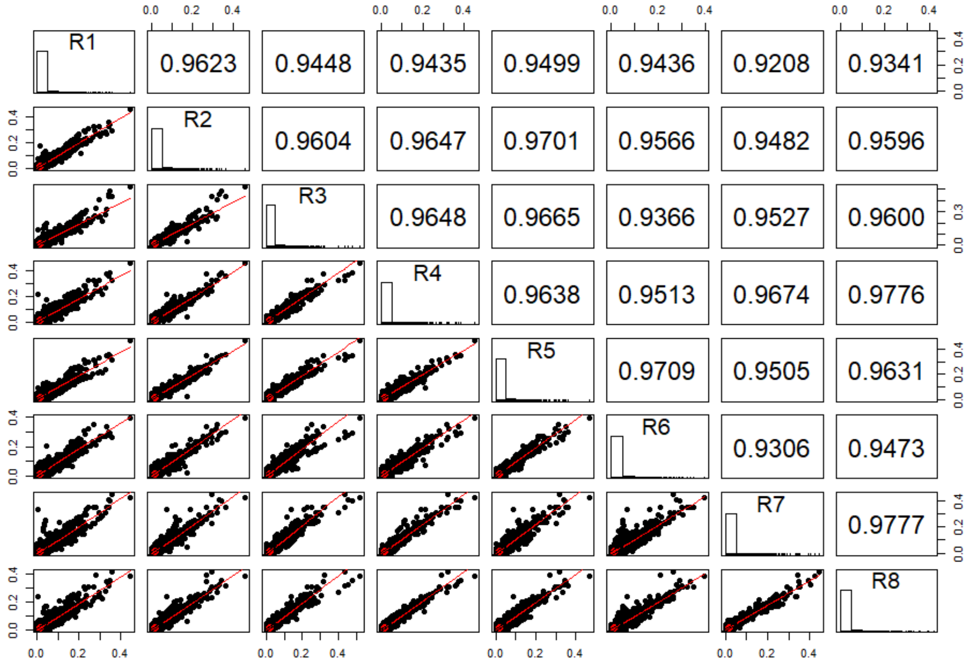

Figure S3. Linear correlation of RNAlater (R) replicates. Plots are displayed in the lower panel and the corresponding Pearson correlation coefficients are displayed in the upper panel.

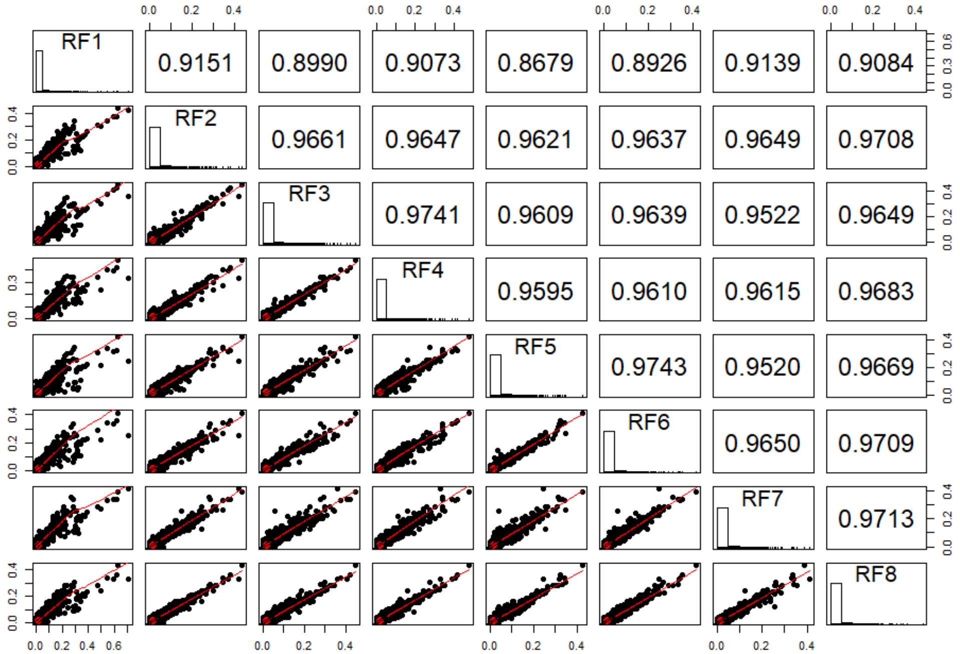

Figure S4. Linear correlation of RNAlater + flash-freezing (RF) replicates. Plots are displayed in the lower panel and the corresponding Pearson correlation coefficients are displayed in the upper panel.

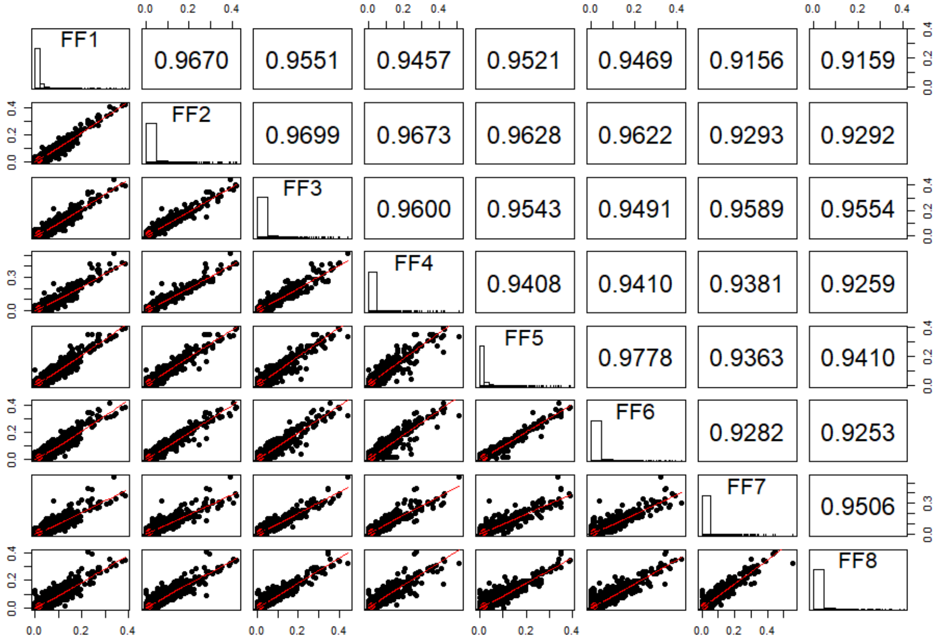

Figure S5. Linear correlation of Flash-frozen (FF) replicates. Plots are displayed in the lower panel and the corresponding Pearson correlation coefficients are displayed in the upper panel.

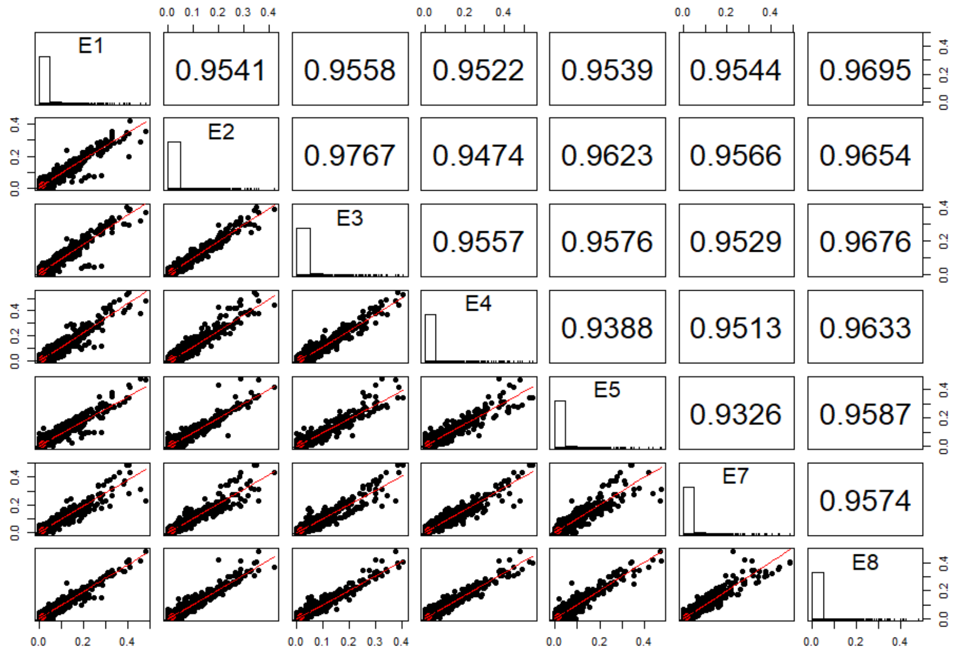

Figure S6. Linear correlation of ethanol (E) replicates. Plots are displayed in the lower panel and the corresponding Pearson correlation coefficients are displayed in the upper panel.

We analyzed the metaproteome-based taxonomic compositions of individual samples using the method described in [3]. Briefly, we filtered proteins to include only those with at least 2 protein unique peptides to increase the confidence in taxonomic identifications, and we summed Peptide Spectrum Matches (PSMs) by taxon; sums were then used to estimate the biomass contribution of each taxon in the metaproteomes. This analysis showed that host proteins contributed 5.7 +/- 1.6% (n = 47) of each metaproteome, while the microbial proteins contributed 94.2 +/- 1.6% (n = 47) and dietary proteins from wheat, the main component of the mice's diet, contributed roughly 0.1 +/- 0.2 % (n = 47). Taxonomic profiles in terms of biomass contribution were consistent across all replicates, with minor variability (as shown in Figure S7). Figures S7 A and S7 B show the representation of the detected phyla in each sample. The phylum Firmicutes was dominant in these samples, making up 86.7 +/- 0.92 % (n = 47) of the total proteinaceous biomass. Microorganisms of unknown taxonomy made up 13.1 +/- 0.92 % (n = 47) of the total proteinaceous biomass of the samples. The number of detected proteins of known taxonomy was higher than expected given that only 67.8% of the microbial protein sequences in the database were assigned a

taxonomy at the phylum level and 9.8% of the microbial sequences were annotated to the genus level [4]. We found that 11.1 +/- 0.53 % (n = 47) of the total proteinaceous biomass in our samples had a taxonomy assigned at the genus level, representing 28 microbial genera. Over half of these genera have very few Peptide Spectrum Matches (PSMs). The most abundant genera detected in the samples included *Clostridium, Eubacterium, Butyrivibrio, Lactobacillus, Turicibacter, Blautia, Roseburia*, and *Coprococcus* (Figure S2 C).

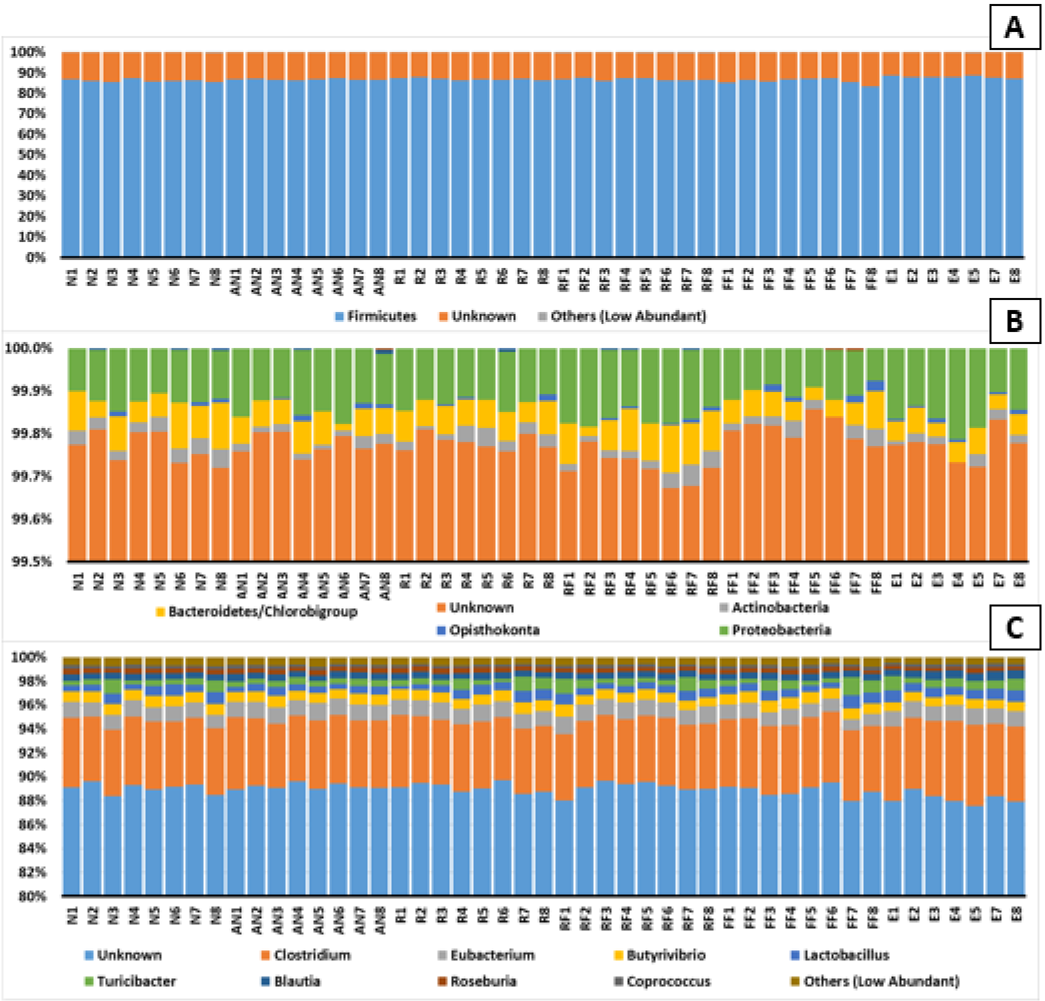

Figure S7: Taxonomic composition of individual samples as proteinaceous biomass contribution. (A) Relative abundances of the major phyla: Firmicutes, Unknown, and Others. “Others” corresponds to the sum of the phyla that were detected with very few Peptide Spectrum Matches (PSMs). The category “Others” made up less than 0.4% of the total biomass contribution of the samples. (B) Relative abundances of the lowly abundant phyla; i.e. composition of the category “others” shown in panel A. (C)

Relative abundances of the most abundant genera. N = NAP buffer; AN = Autoclaved NAP buffer; R = RNAlater; RF = RNAlater + Flash-freezing; FF = Flash-freezing; E = Ethanol.

Table S1: Overview of the metaproteomic data provided with this study (dataset PXD024115 on the ProteomeXchange Consortium).

**Sample name**	**Treatment**	**Storage time**	**Peptide concentration (ng/uL)**	**Peptide load (ng)**	**Gradient length (min)**
AN1	Autoclaved NAP buffer	1 week	542.81	600	140
AN2	Autoclaved NAP buffer	1 week	551.851	600	140
AN3	Autoclaved NAP buffer	1 week	694.574	600	140
AN4	Autoclaved NAP buffer	1 week	366.24	600	140
AN5	Autoclaved NAP buffer	4 weeks	904.853	600	140
AN6	Autoclaved NAP buffer	4 weeks	473.039	600	140
AN7	Autoclaved NAP buffer	4 weeks	703.288	600	140
AN8	Autoclaved NAP buffer	4 weeks	533.189	600	140
E1	95% Ethanol	1 week	261.009	600	140
E2	95% Ethanol	1 week	439.121	600	140
E3	95% Ethanol	1 week	391.333	600	140
E4	95% Ethanol	1 week	143.315	600	140
E5	95% Ethanol	4 weeks	461.439	600	140
E6	95% Ethanol	4 weeks	191.788	600	140
E7	95% Ethanol	4 weeks	329.92	600	140
E8	95% Ethanol	4 weeks	325.898	600	140
FF1	Flash Freezing	1 week	609.099	600	140
FF2	Flash Freezing	1 week	671.558	600	140
FF3	Flash Freezing	1 week	461.606	600	140

FF4	Flash Freezing	1 week	628.12	600	140
FF5	Flash Freezing	4 weeks	283.113	600	140
FF6	Flash Freezing	4 weeks	228.295	600	140
FF7	Flash Freezing	4 weeks	253.664	600	140
FF8	Flash Freezing	4 weeks	237.902	600	140
N1	NAP buffer	1 week	607.684	600	140
N2	NAP buffer	1 week	587.895	600	140
N3	NAP buffer	1 week	599.197	600	140
N4	NAP buffer	1 week	841.462	600	140
N5	NAP buffer	4 weeks	608.211	600	140
N6	NAP buffer	4 weeks	543.518	600	140
N7	NAP buffer	4 weeks	634.832	600	140
N8	NAP buffer	4 weeks	490.3	600	140
R1	RNAlater	1 week	694.166	600	140
R2	RNAlater	1 week	633.792	600	140
R3	RNAlater	1 week	871.938	600	140
R4	RNAlater	1 week	555.872	600	140
R5	RNAlater	4 weeks	649.313	600	140
R6	RNAlater	4 weeks	508.285	600	140
R7	RNAlater	4 weeks	333.275	600	140
R8	RNAlater	4 weeks	563.871	600	140
RF1	RNAlater + Flash Freezing	1 week	665.051	600	140
RF2	RNAlater + Flash Freezing	1 week	413.117	600	140
RF3	RNAlater + Flash Freezing	1 week	375.914	600	140
RF4	RNAlater + Flash Freezing	1 week	521.545	600	140
RF5	RNAlater + Flash Freezing	4 weeks	479.529	600	140
RF6	RNAlater + Flash Freezing	4 weeks	525.108	600	140

RF7	RNAlater + Flash Freezing	4 weeks	390	600	140
RF8	RNAlater + Flash Freezing	4 weeks	568.535	600	140

1. Krogh A, Larsson B, von Heijne G, Sonnhammer ELL. Predicting transmembrane protein topology with a hidden Markov model: application to complete genomes. J Mol Biol. 2001;305:567–80.

2. Zybailov B, Mosley AL, Sardiu ME, Coleman MK, Florens L, Washburn MP. Statistical Analysis of Membrane Proteome Expression Changes in Saccharomyces cerevisiae. J Proteome Res. 2006;5:2339–47.

3. Kleiner M, Thorson E, Sharp CE, Dong X, Liu D, Li C, et al. Assessing species biomass contributions in microbial communities via metaproteomics. Nat Commun. 2017;8:1558.

4. Xiao L, Feng Q, Liang S, Sonne SB, Xia Z, Qiu X, et al. A catalog of the mouse gut metagenome. Nat Biotechnol. 2015;33:1103–8.
