# Supplementary material for: Evaluation of Sample Preservation and Storage Methods for Metaproteomics Analysis of Intestinal Microbiomes
Source: Microbiol Spectr. 2021 Dec 15;9(3):e01877-21. doi: 10.1128/Spectrum.01877-21 (PMC8672883; doi:10.1128/Spectrum.01877-21)
Supplement: Reviewer comments [file reviewer-comments.pdf]

AMERICAN SOCIETY FOR MICROBIOLOGY | Microbiology Spectrum

Evaluation of sample preservation and storage methods for metaproteomics analysis of intestinal microbiomes

Angie Mordant and Manuel Kleiner

Corresponding Author(s): Angie Mordant, North Carolina State University

Review Timeline:

Submission Date:	October 12, 2021
Accepted:	October 31, 2021

Editor: Cezar Khursigara

Reviewer(s): The reviewers have opted to remain anonymous.

Transaction Report:

(Note: With the exception of the correction of typographical or spelling errors that could be a source of ambiguity, letters and reports are not edited. The original formatting of letters and referee reports may not be reflected in this compilation.)

DOI: https://doi.org/10.1128/Spectrum.01877-21

October 31, 2021

Ms. Angie Mordant
North Carolina State University
Plant & Microbial Biology
4510 Thomas Hall
112 Derieux Place
Raleigh, NC 27695

Re: Spectrum01877-21 (Evaluation of sample preservation and storage methods for metaproteomics analysis of intestinal microbiomes)

Dear Ms. Angie Mordant:

Your manuscript has been accepted, and I am forwarding it to the ASM Journals Department for publication. You will be notified when your proofs are ready to be viewed.

The ASM Journals program strives for constant improvement in our submission and publication process. Please tell us how we can improve your experience by taking this quick Author Survey.

As an open-access publication, Spectrum receives no financial support from paid subscriptions and depends on authors' prompt payment of publication fees as soon as their articles are accepted. You will be contacted separately about payment when the proofs are issued; please follow the instructions in that e-mail. Arrangements for payment must be made before your article is published. For a complete list of **Publication Fees**, including supplemental material costs, please visit ourwebsite.

Corresponding authors may join or renew ASM membership to obtain discounts on publication fees. Need to upgrade your membership level? Please contact Customer Service at Service@asmusa.org.

Thank you for submitting your paper to Spectrum.

Sincerely,

Cezar Khursigara
Editor, Microbiology Spectrum

Journals Department
American Society for Microbiology
1752 N St., NW
Washington, DC 20036
E-mail: spectrum@asmusa.org

Supplemental Material: Accept
File S5: Accept
File S3: Accept
File S1: Accept
File S4: Accept
File S2: Accept
